# *Ad hoc* Analysis of the Phase III ENGOT-OV16/NOVA Study: Niraparib Efficacy in Germline *BRCA* Wild-type Recurrent Ovarian Cancer with Homologous Recombination Repair Defects

**DOI:** 10.1158/2767-9764.CRC-22-0240

**Published:** 2022-11-15

**Authors:** Mansoor Raza Mirza, Gabriel Lindahl, Sven Mahner, Andrés Redondo, Michel Fabbro, Bobbie J. Rimel, Jørn Herrstedt, Amit M. Oza, Ulrich Canzler, Jonathan S. Berek, Antonio González-Martín, Phillipe Follana, Rosemary Lord, Masoud Azodi, Kasey Estenson, Zebin Wang, Yong Li, Divya Gupta, Ursula Matulonis, Bin Feng

**Affiliations:** 1 Nordic Society of Gynaecological Oncology (NSGO) and Department of Oncology Rigshospitalet–Copenhagen University Hospital, Copenhagen, Denmark.; 2 Nordic Society of Gynaecological Oncology (NSGO) and Department of Oncology, Linköping University Hospital, Linköping, Sweden.; 3 Arbeitsgemeinschaft Gynaekologische Onkologie Studiengruppe (AGO) and Department of Gynecology and Gynecologic Oncology, University Medical Center Hamburg-Eppendorf, Hamburg, Germany.; 4 Grupo Español de Investigación en Cáncer de Ovario (GEICO) and Department of Medical Oncology, Hospital Universitario La Paz-IdiPAZ, Madrid, Spain.; 5 ICM Val d'Aurelle Parc Euromedecine, Oncologie Médicale, Montpellier, GINECO, Paris, France.; 6 Cedars-Sinai Cancer, Cedars-Sinai Medical Center, Los Angeles, California.; 7 Department of Clinical Oncology and Palliative Care Zealand University Hospital Roskilde and Næstved, University of Copenhagen, Copenhagen, Denmark.; 8 Division of Medical Oncology and Hematology, Princess Margaret Cancer Centre, University Health Network, Toronto, Ontario, Canada.; 9 Arbeitsgemeinschaft Gynaekologische Onkologie Studiengruppe (AGO) and Department of Gynecology and Obstetrics, Medical Faculty and University Hospital Carl Gustav Carus, Technische Universität Dresden, Dresden, Germany.; 10 National Center for Tumor Diseases (NCT), Partner Site Dresden, Dresden, Germany.; 11 Stanford University School of Medicine and Stanford Cancer Institute, Stanford, California.; 12 Medical Oncology Department, Clínica Universidad de Navarra, Madrid, Program in Solid Tumors, Center for Applied Medical Research (CIMA), Pamplona, Spain, Grupo Español de Investigación en Cáncer de Ovario (GEICO), Madrid, Spain.; 13 Centre Antoine Lacassagne, Nice, France.; 14 Medical Oncology, Clatterbridge Cancer Centre NHS Foundation Trust, Bebington, United Kingdom.; 15 Department of Obstetrics, Gynecology and Reproductive Sciences, Yale University School of Medicine, New Haven, Connecticut.; 16 GSK, Philadelphia, Pennsylvania.; 17 GSK, Waltham, Massachusetts.; 18 GSK, Waltham, Massachusetts.; 19 Department of Medical Oncology, Dana-Farber Cancer Institute, Boston, Massachusetts.; 20 Harvard Medical School, Boston, Massachusetts.

## Abstract

In this analysis, we examined the relationship between progression-free survival (PFS) and mutation status of 18 homologous recombination repair (HRR) genes in patients in the non-germline *BRCA*-mutated (non-g*BRCA*m) cohort of the ENGOT-OV16/NOVA trial (NCT01847274), which evaluated niraparib maintenance therapy for patients with recurrent ovarian cancer. This *post hoc* exploratory biomarker analysis was performed using tumor samples collected from 331 patients enrolled in the phase III ENGOT-OV16/NOVA trial's non-g*BRCA*m cohort. Niraparib demonstrated PFS benefit in patients with either somatic *BRCA-*mutated (s*BRCA*m; HR, 0.27; 95% confidence interval, CI, 0.08–0.88) or *BRCA* wild-type (*BRCA*wt; HR, 0.47; 95% CI, 0.34–0.64) tumors. Patients with *BRCA*wt tumors with other non-*BRCA* HRR mutations also derived benefit from niraparib (HR, 0.31; 95% CI, 0.13–0.77), as did patients with *BRCA*wt/HRRwt (HRR wild-type) tumors (HR, 0.49; 95% CI, 0.35–0.70). When patients with *BRCA*wt/HRRwt tumors were further categorized by genomic instability score (GIS), clinical benefit was observed in patients with homologous recombination–deficient (GIS ≥ 42; HR, 0.33; 95% CI, 0.18–0.61) and in patients with homologous recombination–proficient (HRp; GIS < 42; HR, 0.60; 95% CI, 0.36–0.99) disease. Although patients with s*BRCA*m, other non-*BRCA* HRR mutations, or GIS ≥ 42 benefited the most from niraparib treatment, PFS benefit was also seen in HRp (GIS < 42) patients without HRR mutations. These results support the use of niraparib in patients with recurrent ovarian cancer regardless of *BRCA*/HRR mutation status or myChoice CDx GIS.

**Significance:**

We retrospectively evaluated the mutational profile of HRR genes in tumor samples from 331 patients from the non-germline *BRCA*-mutated cohort of the phase III NOVA trial of patients with platinum-sensitive high-grade serous ovarian cancer. Patients with non-*BRCA* HRR mutations generally benefited from second-line maintenance treatment with niraparib compared with placebo.

## Introduction

The PARP family of nuclear proteins is recruited to DNA repair complexes and activated on sensing DNA single-strand breaks (SSB), playing a crucial role in SSB repair (1). In the presence of a PARP inhibitor (PARPi), unrepaired SSBs lead to stalled replication forks and accumulation of DNA double-strand breaks (DSB; ref. 2). In normal cells, DSBs are repaired effectively by a high-fidelity, error-free DNA repair process called homologous recombination repair (HRR; ref. 3). In cells with faulty HRR, called homologous recombination deficient (HRd), such as those bearing *BRCA* mutations (*BRCA*m), PARP inhibition induces accumulation of DNA DSBs, leading to the activation of nonhomologous end-joining pathway, an error-prone process to repair DNA DSBs; this process results in chromosomal instability, cell-cycle arrest, and subsequent apoptotic cell death (3). PARP inhibition also results in PARP–DNA complexes by trapping PARP1/2 protein on the DNA, which will further intensify the DNA replication fork damage. This synthetic lethality between PARP inhibition and homologous recombination defects has served as the basis of PARPi therapy development in multiple solid tumors and is the best-characterized mechanism of action for these agents (4, 5). In addition, preclinical and clinical studies have demonstrated that tumor cells that are homologous recombination proficient (HRp) may also respond to PARPi, suggesting the utility of PARPi beyond HRd tumors (6–9).

In addition to *BRCA1* and *BRCA2*, other genes play critical roles in orchestrating the HRR process, including genes involved in DNA DSB recognition (7), initiation of HRR (7), DNA resection (10), RAD51 filament strand invasion (11, 12), DNA synthesis (12), and Holliday junction resolution (13). Defects in expressing these HRR genes will impair the integrity of HRR and may confer sensitivity to PARP inhibition. The sensitivity of tumors with HRR mutations (HRRm), including *BRCA1, BRCA2, PALB2,* and *RAD51C*, to PARPi has been reported in preclinical research (14), as well as clinically (15, 16), across tumor types (Supplementary Table S1). In ovarian cancer, analysis of samples from the ARIEL2 trial of rucaparib maintenance therapy found *RAD51C* and *RAD51D* mutations as well as high-level *BRCA1* promoter methylation to be associated with PARPi sensitivity (17). However, the reported clinical evidence in ovarian maintenance with niraparib is limited.

The ENGOT-OV16/NOVA trial (NCT01847274) of niraparib maintenance enrolled 553 patients with platinum-sensitive recurrent ovarian cancer who responded to the penultimate platinum-based chemotherapy: 203 in the germline *BRCA*m (g*BRCA*m) (niraparib, *n*  =  138; placebo, *n*  =  65) and 350 in the non-g*BRCA*m (niraparib, *n*  =  234; placebo, *n*  =  116) cohorts (7). Patients in both cohorts experienced a statistically significant improvement in median progression-free survival (mPFS) compared with those in the placebo arm [21.0 vs. 5.5 months in the g*BRCA*m cohort (HR, 0.27; 95% confidence interval, CI, 0.17--0.41) and 9.3 vs. 3.9 months in the overall non-g*BRCA*m cohort (HR, 0.45; 95% CI, 0.34--0.61); *P* < 0.001 for both comparisons] (7). On the basis of the results of the ENGOT-OV16/NOVA trial, niraparib became the first PARPi approved for maintenance treatment of platinum-sensitive recurrent ovarian cancer regardless of biomarker status.

Whereas the NOVA study used the BRAC*Analysis* assay (Myriad Genetics, Inc.) to determine g*BRCA*m status for patient enrollment, the tissue-based myChoice CDx (Myriad Genetics, Inc.) was used to determine tumor, or somatic, *BRCA*m status and genomic instability score (GIS). Patient tumors were identified as HRd [somatic *BRCA*m (s*BRCA*m) and/or GIS ≥  42] or HRp (non-s*BRCA*m and GIS < 42). Patients whose tumors were identified as HRd in the non-g*BRCA*m cohort had longer mPFS in the niraparib arm than in the placebo arm [12.9 vs. 3.8 months (HR, 0.38; 95% CI, 0.24--0.59)]. In addition, patients in the niraparib arm who were identified as HRp had a significantly longer mPFS than those in the placebo arm [6.9 vs. 3.8 months (HR, 0.58; 95% CI, 0.36--0.92)] (7). These results potentially reflect the limitations of current tests to reliably capture patients with genomic scarring and HR-deficient tumors who could potentially benefit from therapy. In addition, they also suggest that mechanisms independent of *BRCA*m or HRd may confer clinical benefit with niraparib PARPi in ovarian cancer.

We performed a comprehensive retrospective analysis using tumor samples collected from the non-g*BRCA*m cohort in the ENGOT-OV16/NOVA trial to explore additional biomarkers or mechanisms that may predict sensitivity to niraparib. The mutation status of the 18 HRR genes, including *BRCA1/2*, within the Myriad HRD research assay (Myriad Genetics, Inc.) was evaluated as a biomarker for niraparib.

## Materials and Methods

### Patients and Samples

Of the 553 patients enrolled in the ENGOT-OV16/NOVA trial, 350 patients were in the non-g*BRCA*m cohort as determined by BRAC*Analysis* (Myriad Genetics, Inc.), which uses blood or saliva samples to test for the presence of deleterious or suspected deleterious germline *BRCA1/2* mutations. Of the 350 patients in the non-g*BRCA*m cohort, 331 had archival tumor samples available for additional tumor biomarker testing and were included in this analysis.

As part of the ENGOT-OV16/NOVA trial, myChoice CDx was performed on tumor samples prior to database lock. The myChoice CDx test is an integrated next-generation sequencing test assessing s*BRCA*m status and measuring tumor genomic instability (18). Three algorithms were used to assess genomic instability—loss of heterozygosity (LOH) profiles, telomeric allelic imbalance, and large-scale state transitions—resulting in the myChoice CDx GIS, which is the sum of the three individual scores (Supplementary Fig. S1). Although the myChoice CDx GISs distribute along a continuum, patients were categorized as either myChoice CDx HRD-positive (now called HRd) or HRD-negative (now called HRp) according to the prespecified cut-off score of 42 and/or s*BRCA*m presence (19). This analysis excludes patients who were enrolled in the g*BRCA*m cohort of the trial, as determined by BRAC*Analysis*, regardless of HR status.

### HRR Biomarker Test

The Myriad research-grade assay (Myriad Genetics, Inc.) was performed on the 331 available patient tumor samples from the 350 patients in the non-g*BRCA*m cohort to determine the mutation status of 18 HRR genes (*ATM, ATR, BAP1, BARD1, BRCA1, BRCA2, BRIP1, MRE11A, NBN, PALB2, RAD50, RAD51B, RAD51C, RAD51D, RAD54B, RAD54L, XRCC2,* and *XRCC3*). Deleterious or suspected deleterious mutations were defined by Myriad Genetics based on review of multiple lines of evidence.

### Statistical Analysis

All statistical analyses in this article were *post hoc*. An exploratory analysis was performed on the 331 patients from the non-g*BRCA*m cohort with available tumor samples to determine HRR gene mutation status. For the subgroup analyses, we performed a two-sided log-rank test using the stratification factors from randomization (best response to last platinum-based therapy, HRD status, and time from penultimate platinum-based therapy to progression) to analyze PFS, which was summarized using Kaplan--Meier methods. Patients were censored according to the methods used in the primary analysis (7). We estimated HRs with two-sided 95% CIs using a stratified Cox proportional hazards model with the stratification factors used in randomization.

HRs refer to the comparison of the niraparib arm with the placebo control arm. Formal *P*-value correction for multiple testing was not applied, but the multiplicity was accounted for in the interpretation of results. This approach was considered the most suitable given the exploratory nature of the analyses and that the measured biomarkers (*BRCA*m, HRRm, and myChoice CDx GIS) were selected on the basis of biological relevance.

### Data Availability

All patients provided written informed consent as approved by an Institutional Review Board, in accordance with ethical guidelines as described in the U.S. Common Rule. As patients were not specifically consented for open-access genomic data, complete sequencing data, such as BAM files, cannot be shared publicly. Anonymized individual participant data and study documents can be requested for further research from www.clinicalstudydatarequest.com.

## Results

### *Post hoc* Classification of HRR Gene Mutation Status Among Patients in the Non-g*BRCA*m Cohort of the Phase III ENGOT-OV16/NOVA Trial

Baseline characteristics of the 331 patients from non-g*BRCA*m cohort with known HRR status were included in this analysis are shown in Supplementary Table S2. Demographic and clinical characteristics were well balanced in the niraparib and placebo arms. For exploratory purposes, patients whose tumor contained a loss-of-function (LOF) mutation in at least one of the 18 HRR genes including *BRCA1/2* were considered as HRRm (Supplementary Fig. S1). Of the 331 tumor samples analyzed from the non-g*BRCA*m cohort, 283 (85.5%, 283/331) were *BRCA* wild type (*BRCA*wt) with no detectable deleterious or suspected deleterious mutation in *BRCA1/2,* and 48 (14.5%, 48/331) carried deleterious or suspected deleterious somatic *BRCA1/2* mutations (s*BRCA*m; Supplementary Fig. S2A). Of the 48 patients with s*BRCA*m disease, 43 carried biallelic s*BRCA*m (*BRCA*m and LOH event), 1 carried a monoallelic *BRCA*m, and 4 carried *BRCA*m with unknown allelic status of the tumor (Supplementary Fig. S2B). Of the 283 patients in the *BRCA*wt subgroup, 41 (14.5%, 41/283) had an LOF mutation in at least 1 non-*BRCA* HRR gene (non-*BRCA* HRRm; Supplementary Fig. S2A). Among the 16 non-*BRCA* HRR genes evaluated in this cohort, *RAD51C* was the most commonly mutated (*n* =  9), followed by *BRIP1* (*n* =  7), and the remaining genes had mutations observed in 5 or fewer patients. None of the tested samples had mutated *RAD54B* or *XRCC2* (Supplementary Fig. S2A). When using OncoPrint plot to illustrate the mutational spectrum at the patient level by different treatment groups, LOF mutations in any of the other 16 non-*BRCA* HRR genes were rarely detected (2/48) in the s*BRCA*m tumor samples (Supplementary Fig. S2B). One patient had both *BRCA1* and *RAD54B* mutations, and 1 patient had both *BRCA2* and *RAD54 L* mutations. Among patients with non-*BRCA* HRRm tumors, only 1 patient had both *RAD51C* and *BRIP1* mutation (Supplementary Fig. S2B).

### Relationships Between GIS, *BRCA* Mutation Status, and Non-*BRCA* HRRm Status

**FIGURE 1:**
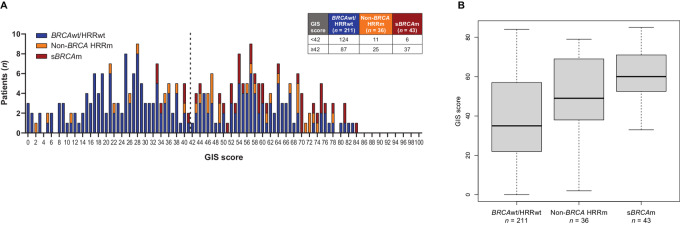
HRR status in the non-g*BRCA*m cohort of NOVA. **A,** GIS distribution by biomarker. **B,** GIS by biomarker. Of the 331 patients with HRR results, 41 did not have a GIS available for analysis; 2 patients had both *BRCA* mutation and non-*BRCA* HRRm and were classified as s*BRCA*m. *BRCA*wt, *BRCA* wild type; *gBRCA*m, germline *BRCA* mutated; GIS, genomic instability score; HRR, homologous recombination repair; HRRm, homologous recombination repair mutated; HRRwt, homologous recombination repair wild type; *sBRCA*m, somatic *BRCA* mutated.

The GIS histogram of the non-g*BRCA*m cohort showed a bimodal distribution, and the current Myriad GIS cutoff of 42 separated the two modes (Fig. 1A). All 43 s*BRCA*m samples with myChoice CDx GISs available for analysis had scores ≥33, and 37 had a GIS ≥ 42 (Fig. 1A). myChoice CDx GISs for non-*BRCA* HRRm samples ranged from 2 to 79. Of the 36 patients with non-*BRCA* HRRm tumors with available GIS, 25 had a GIS ≥ 42 (Fig. 1A). s*BRCA*m tumors had the highest median GIS (Fig. 1B), non-*BRCA* HRRm tumors had an intermediate median GIS, and *BRCA*wt/HRR wild-type (HRRwt) tumors had the lowest median GIS (Fig. 1B).

### Efficacy by s*BRCA*m Status Among Patients in the Non-gBRCAm Cohort

**FIGURE 2:**
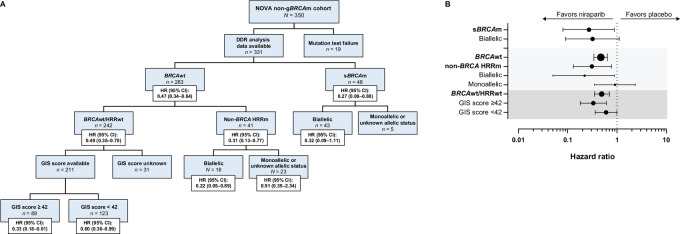
Efficacy by biomarker subgroup in the non-g*BRCA*m cohort presented by biomarker allocation flowchart (**A**) and forest plot (**B**). *BRCA*wt, *BRCA* wild type; DDR, DNA damage repair; g*BRCA*m, germline *BRCA* mutated; GIS, genomic instability score; HR, hazard ratio; HRRm, homologous recombination repair mutated; HRRwt, homologous recombination repair wild type; s*BRCA*m, somatic *BRCA* mutated.

**FIGURE 3:**
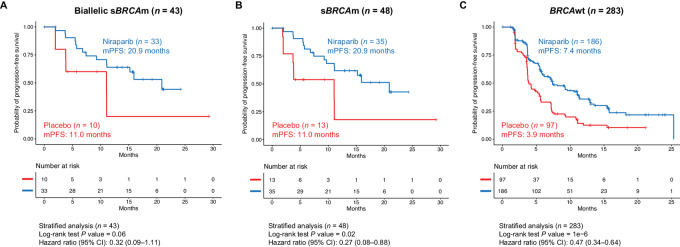
Clinical benefit of niraparib in patients with s*BRCA*m versus patients with *BRCA*wt tumors in the NOVA non-g*BRCA*m cohort. Kaplan--Meier estimates of progression-free survival in the niraparib group and in the placebo group among patients with biallelic s*BRCA*m (**A**), s*BRC*Am (**B**), and *BRCA*wt tumors (**C**). *BRCA*wt, *BRCA* wild type; g*BRCA*m, germline *BRCA* mutated; mPFS, median progression-free survival; s*BRCA*m, somatic *BRCA* mutated.

Exploratory *post hoc* analysis of mPFS was performed to evaluate the benefit of niraparib in patients with s*BRCA*m (Figs. 2 and 3). The HR of niraparib versus placebo was 0.32 (95% CI, 0.09--1.11; mPFS, 20.9 vs. 11.0 months, Δ9.9 months) in the 43 patients with biallelic s*BRCA*m (Figs. 2 and 3A), 0.27 (95% CI, 0.08--0.88; mPFS, 20.9 vs. 11.0 months; Δ9.9 months) in the 48 patients with s*BRCA*m (Figs. 2 and 3B), and 0.47 (95% CI, 0.34--0.64; mPFS, 7.4 vs. 3.9 months, Δ3.5 months) in the 283 patients with *BRCA*wt tumors (Figs. 2 and 3C).

### Efficacy by HRRm Status Among Patients with *BRCA*wt Tumors

**FIGURE 4:**
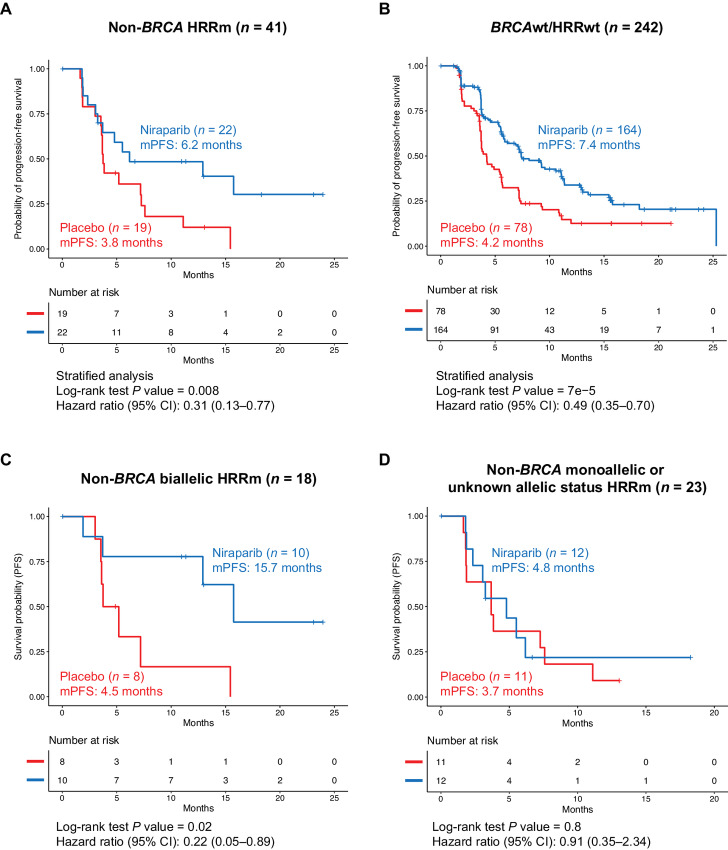
Clinical benefit of niraparib in patients with non-*BRCA* HRRm versus patients with *BRCA*wt/HRRwt tumors in the NOVA non-g*BRCA*m cohort. Kaplan--Meier estimates of PFS in the niraparib group and in the placebo group among patients with non-*BRCA* HRRm (**A**), *BRCA*wt/HRRm (**B**), non-*BRCA* biallelic HRRm (**C**), and non-*BRCA* monoallelic or unknown allelic status HRRm tumors (**D**). *BRCA*wt, *BRCA* wild type; g*BRCA*m, germline *BRCA* mutated; HRRm, homologous recombination repair mutated; HRRwt, homologous recombination repair wild type; mPFS, median progression-free survival.

To evaluate whether other HRRms contributed to the clinical benefit of niraparib observed in patients with *BRCA*wt tumors, an exploratory *post hoc* analysis of mPFS was performed in subgroups of patients with or without other HRRms in *BRCA*wt tumors. The HR of niraparib versus placebo was 0.31 (95% CI, 0.13--0.77; mPFS, 6.2 vs. 3.8 months, Δ2.4 months) in the 41 patients with non-*BRCA*m HRRm tumors (Fig. 4A) and 0.49 (95% CI, 0.35--0.70; 7.4 vs. 4.2 months, Δ3.2 months) in the 242 patients with *BRCA*wt/HRRwt tumors (Fig. 4B). This result suggests that mutations in non-*BRCA* HRR genes may impair HRR and sensitize patients to niraparib beyond *BRCA1/2*, demonstrating similar positive predictive value to that of s*BRCA* mutation. Nevertheless, patients with *BRCA*wt tumors, regardless of their HRR gene mutation status, also benefit from treatment with niraparib. The positive predictive value of mutations in at least one of the 16 HRR genes is similar to that of s*BRCA*m, as suggested by HRs of biomarker-positive cohorts, *BRCA*wt/*HRR*m (HR, 0.31; 95% CI, 0.13--0.77) and s*BRCA*m (HR, 0.27; 95% CI, 0.08--0.88), when comparing between the niraparib and placebo arms (Figs. 2–4); however, neither biomarker demonstrates optimal negative predictive value, as suggested by HRs of biomarker negative cohorts, *BRCA*wt/*HRR*wt (HR, 0.49; 95% CI, 0.35--0.70) and *BRCA*wt (HR, 0.47; 95% CI, 0.34--0.64) when comparing between the niraparib and placebo arms (Figs. 2–4). When comparing niraparib and placebo, HRs of the biomarker-positive cohorts (*BRCA*wt/*HRR*m and s*BRCA*m, respectively) are smaller than HRs of the biomarker-negative cohorts (*BRCA*wt/*HRR*wt and *BRCA*wt, respectively), but HRs of both biomarker-negative cohorts are still statistically significant and less than 1.

In a subanalysis of the 41 patients with non-*BRCA* HRRm tumors, the HR of niraparib versus placebo was 0.22 (95% CI, 0.05--0.89; mPFS, 15.7 vs. 4.5 months, Δ11.2 months) in the 18 patients with biallelic HRRm (Fig. 4C) and 0.91 (95% CI, 0.35--2.34; mPFS, 4.8 vs. 3.7 months, Δ1.2 months) in the 23 patients whose HRRms were monoallelic or had unknown allelic status (Fig. 4D). Because non-*BRCA* HRR genes function differently within the HRR pathway, niraparib treatment response was also evaluated in patients with tumor mutations in the more well-characterized HRR genes known to contribute to HRD and PARPi sensitivity (i.e., *RAD51C*, *RAD51D*, *BRIP1*, and *PALB2*) and patients with other, less well-studied HRR genes. Although the data should be approached with caution because of the small sample size, the benefit of niraparib treatment was more apparent in patients with mutations in well-known HRR genes than in the other group (Supplementary Table S3).

### Analysis of HRRms Against GISs Among Patients in the NOVA Trial

**FIGURE 5:**
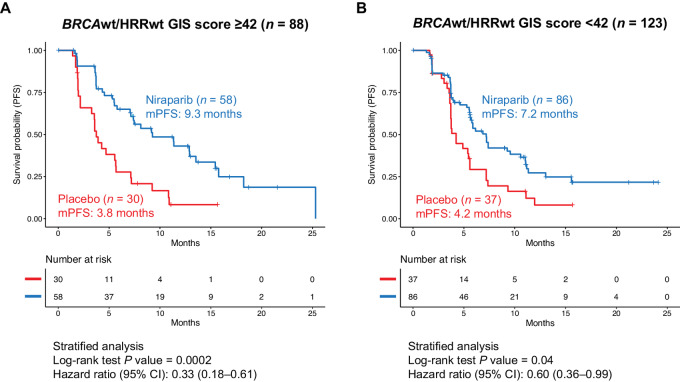
Clinical benefit of niraparib in patients with *BRCA*wt/HRRwt tumors with GIS ≥ 42 versus <42 in NOVA non-g*BRCA*m cohort. Kaplan--Meier estimates of PFS in the niraparib group and in the placebo group among patients with *BRCA*wt/HRRwt GIS ≥ 42 (**A**) and *BRCA*wt/HRRwt GIS < 42 (**B**) tumors. *BRCA*wt, *BRCA* wild type; g*BRCA*m, germline *BRCA* mutated; GIS, genomic instability score; HRRwt, homologous recombination repair wild type; mPFS, median progression-free survival.

A further exploratory *post hoc* analysis of mPFS was performed to evaluate the predictive value of GIS, with 42 as the cutoff, in patients with *BRCA*wt/*HRR*wt tumors. Niraparib treatment improved mPFS in the 88 patients with a GIS ≥ 42 (HR, 0.33; 95% CI, 0.18--0.61; mPFS, 9.3 vs. 3.8 months, Δ5.5 months; Fig. 5A). Similarly, an improvement in mPFS was seen with niraparib treatment in the 123 patients whose GIS was <42 [niraparib vs. placebo: HR, 0.60 (95% CI, 0.36--0.99); mPFS, 7.2 vs. 4.2 months, Δ3.0 months; Fig. 5B]. Outcomes were also examined by GIS in patients with non-*BRCA* HRRm tumors; however, the sample sizes for each subgroup were too small to draw conclusions (Supplementary Table S4).

## Discussion

We conducted a comprehensive retrospective analysis of the phase III NOVA/ENGOT-OV16 non-g*BRCA*m cohort, evaluating niraparib efficacy in subgroups of patients across the spectrum of biomarker status, including all combinations of s*BRCA* status, with or without a HRRm, and myChoice CDx--identified GIS status. We observed a statistically significant and clinically meaningful benefit of niraparib treatment in the broad NOVA patient population regardless of the biomarker status. These results are consistent with results from the NOVA clinical trial and demonstrate a continuum of benefits across biomarkers, with the highest sensitivity in patients with deleterious *BRCA*m, followed by those with myChoice CDx HRd tumors, and those with myChoice CDx HRp tumors (7, 20). Although HRRm in *BRCA*wt tumors was associated with higher sensitivity to niraparib, we were unable to determine any biomarker that could identify a patient subset that did not show a clinical benefit. Given these results, although the myChoice CDx GIS may help to estimate the magnitude of benefit from maintenance treatment with niraparib, the benefit-risk ratio of this testing is low in this patient population, especially when the failure rate of the test is high (17% in the ENGOT-OV16/NOVA trial).

In this analysis, assessment of the HRR gene mutation status in NOVA revealed several interesting findings. At the patient level, evaluation of the mutational spectrum found that LOF mutations in any of the other 16 non-*BRCA* HRR genes were rare in s*BRCA*m tumor samples, potentially indicating a mutual exclusivity between *BRCA* and other HRRms in some cases. In terms of the relationship between HRRm status and GIS, 25 of 36 patients with non-*BRCA* HRRm tumors had a GIS ≥ 42. This observation is consistent with previous findings from Study 19 (21) and might be explained by the myChoice CDx GIS cutoff of 42, which captures 95% of *BRCA1/2* mutations in breast and ovarian cancer. In patients with non-*BRCA* HRRm tumors, the results suggest that patients with biallelic HRRms may show higher sensitivity to niraparib. This finding is consistent with the hypothesis that most non-*BRCA* HRR genes would require only one intact copy to be functional, which is similar to *BRCA1/2*.

Mechanistically, the responses seen in patients with *BRCA*wt/HRRwt tumors could be potentially explained in several ways. *BRCA* and HRR mutational profiling does not capture promoter methylation. Promoters of *BRCA1* and *RAD51C* are frequently methylated in ovarian cancer, which results in a “BRCAness” phenotype that confers sensitivity to PARPis. *BRCA1* and *RAD51C* promoter methylations are often reported to be mutually exclusive with mutation events (22). Alternatively, the efficacy of niraparib could be explained via a DNA repair--independent effect. PARP1/2 are known to have pleotropic effects that extend beyond DNA repair, and there is growing evidence that PARP-mediated actions impact tumor cell proliferation and viability via alternative mechanisms of action such as PARP-regulated gene transcription (23), ribosome biogenesis (24), and immune activation (25). The efficacy seen with niraparib in *BRCA*wt/HRRwt/HRp tumors could be the result of a functional HR deficiency not identified by either the *BRCA*/HRR mutational analysis or the myChoice CDx HRD test. The myChoice CDx assay is based on capturing genomic scarring resulting from past HR deficiency events, which may not always reflect the current homologous recombination status of the tumor (26). In this analysis, the inability to discern between past genomic scarring and current functional homologous recombination deficiency status could also have been exacerbated by the archival nature of the tumor samples used for testing. The archival samples may not have been reflective of the homologous recombination status of the tumor at the time of niraparib treatment. Moreover, myChoice CDx genomic scarring is based on large structural chromosomal instabilities that do not include the additional genomic features associated with homologous recombination deficiency, such as mutational signatures and microhomology deletion.

When the NOVA trial was designed, the myChoice CDx test was not an approved diagnostic test, and BRAC*Analysis* was used to assign cohorts during randomization. A key difference between BRAC*Analysis* and myChoice CDx is that BRAC*Analysis* only detects germline mutations in *BRCA,* whereas the *BRCA* portion of myChoice CDx detects both germline and somatic mutations (in addition to the genomic scarring score). Therefore, it was not surprising that a number of patients with s*BRCA*m were detected in this *post hoc* analysis. The results from a randomized phase III trial of niraparib in patients with newly diagnosed ovarian cancer (PRIMA/ENGOT-OV26/GOG-3012) were published recently (6). PRIMA used the myChoice CDx test during randomization, and therefore patients with s*BRCA*m have been identified and classified as *BRCA*m and included in the HRd subgroup of that trial. We would expect the results from this analysis—that even patients with GIS < 42 and no known HRR gene mutations benefit from niraparib as maintenance treatment—to be true for PRIMA, and that <1% of patients classified as HRp in the PRIMA trial would have a biallelic *BRCA*m (germline or somatic), consistent with the known parameters of the myChoice CDx test and the results from this analysis. In PRIMA, patients with HRd disease had a HR of 0.43 (95% CI, 0.31--0.59), and patients with HRp disease had a HR of 0.68 (95% CI, 0.49--0.94; ref. 6).

There are some important limitations of these results. This was an exploratory *post hoc* analysis and was not designed or powered to draw definitive conclusions on this topic. All HRR gene mutations are not expected to contribute equally to HR pathway deficiency. However, because the number of patients with any given HRR gene mutation was small, it was not feasible to assess the potential impact of each gene individually. The heterogeneous nature of non-*BRCA* HRR genes also may have contributed to the large 95% CI observed for HRs for patients with non-*BRCA* HRRm tumors. In addition, the myChoice CDx also has its limitations and may have failed to identify all patients with HR-deficient status. Small sample sizes must also be taken into consideration for the different subgroup analyses. Because the number of patients in each subgroup was small and not always evenly distributed between treatment arms, the 95% CIs for the HRs for several subgroups were quite large. Accordingly, caution should be used when extrapolating results to other patient populations. In addition, NOVA only enrolled patients who were platinum sensitive to their penultimate platinum-containing regimen. Because the platinum-free treatment interval following first-line treatment is an important predictor of responsiveness to subsequent treatment (27, 28), the selection of likely responders in NOVA may limit the generalizability of the HRR findings. Future studies to prospectively test the impact of HRR gene mutations on PARPi efficacy (generally and on a per-gene basis) will be important to validate these results.

The results presented here demonstrate the continuum of niraparib efficacy across the different biomarkers in the NOVA study. Patients with s*BRCA*m, other *BRCA* HRRms, or HRD score ≥42 benefitted the most from niraparib treatment. However, significant PFS benefit was also seen in HRp (HRD score <42) patients without HRRms. The results presented here support the use of niraparib in patients with recurrent ovarian cancer regardless of *BRCA*/HRRm status or myChoice CDx GIS, as all studied subgroups demonstrated a clinical benefit with niraparib when compared with placebo.

## Supplementary Material

Supplementary Data S1 Supplementary Data
