# Supplementary material for: Ad hoc Analysis of the Phase III ENGOT-OV16/NOVA Study: Niraparib Efficacy in Germline BRCA Wild-type Recurrent Ovarian Cancer with Homologous Recombination Repair Defects
Source: Cancer Res Commun. 2022 Nov 15;2(11):1436–44. doi: 10.1158/2767-9764.CRC-22-0240 (PMC10035404; doi:10.1158/2767-9764.CRC-22-0240)
Supplement: Supplementary Data S1 — Supplementary Data [file crc-22-0240-s01.docx]

# Supplemental Materials

Supplementary Table S1. HRR genes identified in literature

| **TCGA (1)** | **Preclinical** | **Clinical evidence** | **ARIEL3 (2)** | **TOPARP (3)** |
| --- | --- | --- | --- | --- |
|  | *ATM* (4-6) | *ATM* (3,7) | *ATM* | *ATM* |
|  | *ATR* (6,8) |  | *ATR* |  |
|  | *BAP1* (9) |  |  |  |
| *BARD1* |  |  | *BARD1* |  |
| *BLM* | *BLM* (10) |  | *BLM* |  |
| *BRCA1* |  | *BRCA1* (3) | *BRCA1* | *BRCA1* |
| *BRCA2* |  | *BRCA2* (3) | *BRCA2* | *BRCA2* |
| *BRIP1* |  |  | *BRIP1* |  |
| *EME1* |  |  |  |  |
| *GEN1* |  |  |  |  |
| *MRE11A* |  |  |  |  |
|  | MRE11 (11,12) |  | *MRE11* | *MRE11* |
| *MUS81* |  |  |  |  |
| *NBN* |  |  | *NBN* | *NBN* |
|  | *NBS1* (6,11,13) |  |  |  |
| *PALB2* | *PALB2* (14,15) | *PALB2* (16) | *PALB2* | *PALB2* |
| *RAD50* |  | *RAD50* (17) | *RAD50* |  |
| *RAD51* | *RAD51* (6,18) |  | *RAD51* | *RAD51* |
|  |  |  | *RAD51B* |  |
|  | *RAD51C* (19) |  | *RAD51C* |  |
|  | *RAD51D* (20) |  | *RAD51D* |  |
| *RAD52* | *RAD52* (10) |  | *RAD52* |  |
|  | *RAD54* (6,10) |  | *RAD54L* |  |
| *RBBP8* | *RBBP8* (21,22) |  |  |  |
| *SHFM1* | *SHFM1* (6) |  |  |  |
| *SLX1A* |  |  |  |  |
| *TOP3A* |  |  |  |  |
| *XRCC2* | *XRCC2* (23) |  |  |  |
| *XRCC3* | *XRCC3* (10,23) |  |  |  |
| Abbreviations: HRR, homologous recombination repair; TCGA, The Cancer Genome Atlas. | | | | |

Supplementary Table S2. Baseline characteristics of patients in NOVA with known HRR result

| **Characteristic** | **Non-g*BRCA*m with known HRR result (N = 331)** | |
| --- | --- | --- |
|  | **Niraparib** | **Placebo** |
|  | **(*n* = 221)** | **(*n* =** **110)** |
| **Age, years** | | |
| Median | 63 | 62 |
| (min, max) | (33, 83) | (34, 82) |
| **Weight, kg** | | |
| Median | 66.8 | 66.6 |
| (min, max) | (43.9, 125.7) | (48, 99.5) |
| **Eastern Cooperative Oncology Group performance status, n (%)** | | |
| 0 | 151 (68.3) | 76 (69.1) |
| 1 | 70 (31.7) | 34 (30.9) |
| **Primary tumor site, n (%)** | | |
| Ovarian | 180 (81.4) | 92 (83.6) |
| Primary peritoneal | 23 (10.4) | 8 (7.3) |
| Fallopian tube | 18 (8.1) | 10 (9.1) |
| **Best response to most recent platinum therapy, n (%)** | | |
| Complete | 112 (50.7) | 57 (51.8) |
| Partial | 109 (49.3) | 53 (48.2) |
| **Time to progression after penultimate platinum therapy, n (%)** | | |
| 6 to <12 mo | 134 (60.6) | 67 (60.9) |
| ≥12 mo | 87 (39.4) | 43 (39.1) |
| **Previous bevacizumab use, n (%)** | 60 (27.1) | 28 (25.5) |

Abbreviations: g*BRCA*m, germline *BRCA* mutation; HRR, homologous recombination repair.

Supplementary Table S3. Median PFS in patients with tumors with non-BRCA HRR mutations

|  | **Niraparib** | | **Placebo** | |
| --- | --- | --- | --- | --- |
|  | *n* | mPFS (95% CI), months | *n* | mPFS (95% CI), months |
| **Group 1^a^** |  |  |  |  |
| Biallelic | 9 | 15.7 (12.9–NR) | 4 | 7.2 (3.8–NR) |
| Monoallelic | 2 | NR | 1 | 7.26 |
| **Group 2^b^** |  |  |  |  |
| Biallelic | 1 | NR | 4 | 3.6 (3.0–NR) |
| Monoallelic | 10 | 4.0 (2.3–NR) | 10 | 3.7 (1.8–NR) |

^a^Mutations in *RAD51C*, *RAD51D*, *PALB2.*

^b^Mutations in *ATM*, *ATR*, *BAP1*, *BARD1*, *BRIP1*, *MRE11A*, *NBN*, *RAD50*, *RAD51B*, *RAD54B*, *RAD54L*, *XRCC2*, *XRCC3*.

Abbreviations: HRR, homologous recombination; mPFS, median progression-free survival; NR, not reached; PFS, progression-free survival.

Supplementary Table S4. Median PFS by GIS score in patients with tumors with non-BRCA HRR mutations

|  | **Niraparib** | | **Placebo** | |
| --- | --- | --- | --- | --- |
|  | *n* | mPFS (95% CI), months | *n* | mPFS (95% CI), months |
| GIS score <42 | 6 | 6.2 (4.8–NR) | 5 | 3.8 (3.7–NR) |
| GIS score ≥42 | 12 | 5.5 (3.1–NR) | 13 | 3.8 (3.0–NR) |

Abbreviations: GIS, genomic instability score; HRR, homologous recombination; mPFS, median progression-free survival; NR, not reached; PFS, progression-free survival.

Supplementary Figure S1. HRR genes and HRD status


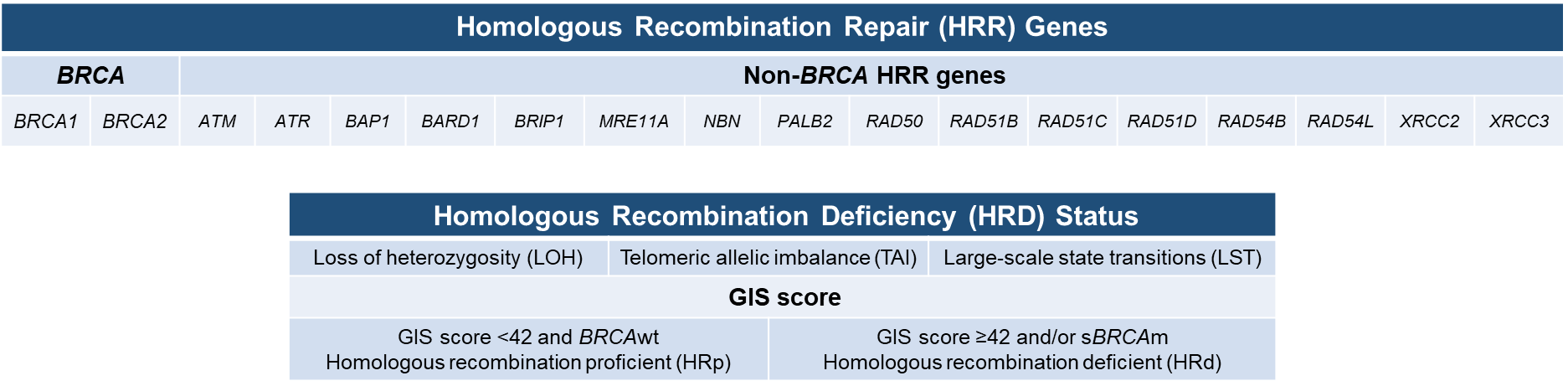


*BRCA*wt, *BRCA* wild type; GIS, genome instability score; s*BRCA*m, somatic *BRCA* mutation.

Supplementary Figure S2. Deleterious or suspected deleterious mutations of HRR genes among the 331 samples tested


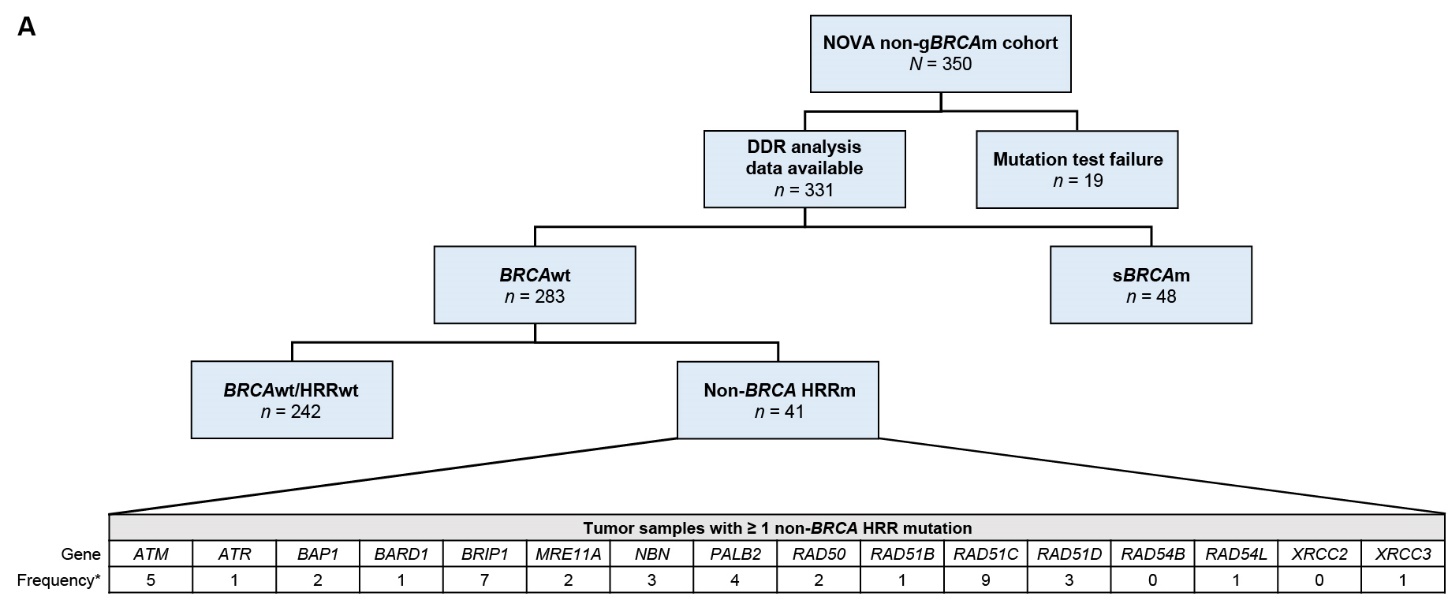


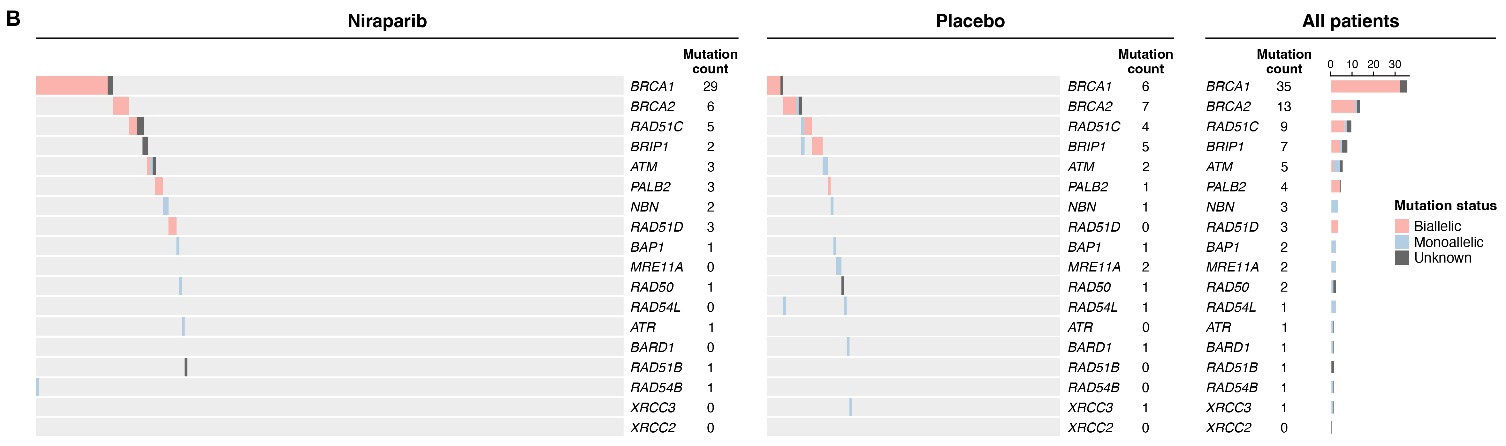


**Figure S2.** Deleterious or suspected deleterious mutations of HRR genes among the 331 samples tested. **A,** Biomarker allocation of the non-g*BRCA* cohort. **B,** OncoPrint plot of the mutational spectrum by treatment arm. Each column represents 1 patient from whom tumor was sequenced. Percentages represent the number of patients with a known or suspected deleterious HRR mutation (numerator) out of 331 (denominator) × 100 for each gene. * Three patients had mutations in 2 HRR genes: 1 patient each had mutations in both *BRCA1* and *RAD54B*, *BRCA2* and *RAD54L*, and *RAD51C* and *BRIP1*.

Abbreviations: *BRCA*m, *BRCA* mutated; *BRCA*wt, *BRCA* wild type; DDR, DNA damage repair; g*BRCA*m, germline *BRCA* mutation; HRR, homologous recombination repair; s*BRCA*m, somatic *BRCA* mutation.

## References for Supplemental Materials

1. Knijnenburg TA, Wang L, Zimmermann MT, Chambwe N, Gao GF, Cherniack AD, et al. Genomic and molecular landscape of DNA damage repair deficiency across The Cancer Genome Atlas. Cell Rep 2018;23(1):239–54 e6.

2. O'Malley DM, Coleman RL, Oza AM, Lorusso D, Aghajanian C, Oaknin A, et al. Results from the phase 3 study ARIEL3: mutations in non-*BRCA* homologous recombination repair genes confer sensitivity to maintenance treatment with the PARP inhibitor rucaparib in patients with recurrent platinum-sensitive high-grade ovarian carcinoma [abstract]. Mol Cancer Ther 2018;17(Suppl 1):LB-A12.

3. Mateo J, Carreira S, Sandhu S, Miranda S, Mossop H, Perez-Lopez R, et al. DNA-repair defects and olaparib in metastatic prostate cancer. N Engl J Med 2015;373(18):1697–708.

4. Wang C, Jette N, Moussienko D, Bebb DG, Lees-Miller SP. ATM-deficient colorectal cancer cells are sensitive to the PARP inhibitor olaparib. Transl Oncol 2017;10(2):190–6.

5. Gilardini Montani MS, Prodosmo A, Stagni V, Merli D, Monteonofrio L, Gatti V, et al. ATM-depletion in breast cancer cells confers sensitivity to PARP inhibition. J Exp Clin Cancer Res 2013;32:95.

6. McCabe N, Turner NC, Lord CJ, Kluzek K, Bialkowska A, Swift S, et al. Deficiency in the repair of DNA damage by homologous recombination and sensitivity to poly(ADP-ribose) polymerase inhibition. Cancer Res 2006;66(16):8109–15.

7. Bang YJ, Im SA, Lee KW, Cho JY, Song EK, Lee KH, et al. Randomized, double-blind phase II trial with prospective classification by ATM protein level to evaluate the efficacy and tolerability of olaparib plus paclitaxel in patients with recurrent or metastatic gastric cancer. J Clin Oncol 2015;33(33):3858–65.

8. Kim H, George E, Ragland R, Rafial S, Zhang R, Krepler C, et al. Targeting the ATR/CHK1 axis with parp inhibition results in tumor regression in BRCA-mutant ovarian cancer models. Clin Cancer Res 2017;23(12):3097–108.

9. Pena-Llopis S, Vega-Rubin-de-Celis S, Liao A, Leng N, Pavia-Jimenez A, Wang S, et al. BAP1 loss defines a new class of renal cell carcinoma. Nat Genet 2012;44(7):751–9.

10. Gottipati P, Vischioni B, Schultz N, Solomons J, Bryant HE, Djureinovic T, et al. Poly(ADP-ribose) polymerase is hyperactivated in homologous recombination-defective cells. Cancer Res 2010;70(13):5389–98.

11. Daemen A, Wolf DM, Korkola JE, Griffith OL, Frankum JR, Brough R, et al. Cross-platform pathway-based analysis identifies markers of response to the PARP inhibitor olaparib. Breast Cancer Res Treat 2012;135(2):505–17.

12. Koppensteiner R, Samartzis EP, Noske A, von Teichman A, Dedes I, Gwerder M, et al. Effect of MRE11 loss on PARP-inhibitor sensitivity in endometrial cancer in vitro. PLoS One 2014;9(6):e100041.

13. Oplustilova L, Wolanin K, Mistrik M, Korinkova G, Simkova D, Bouchal J, et al. Evaluation of candidate biomarkers to predict cancer cell sensitivity or resistance to PARP-1 inhibitor treatment. Cell Cycle 2012;11(20):3837–50.

14. Buisson R, Dion-Cote AM, Coulombe Y, Launay H, Cai H, Stasiak AZ, et al. Cooperation of breast cancer proteins PALB2 and piccolo BRCA2 in stimulating homologous recombination. Nat Struct Mol Biol 2010;17(10):1247–54.

15. Smith MA, Hampton OA, Reynolds CP, Kang MH, Maris JM, Gorlick R, et al. Initial testing (stage 1) of the PARP inhibitor BMN 673 by the pediatric preclinical testing program: PALB2 mutation predicts exceptional in vivo response to BMN 673. Pediatr Blood Cancer 2015;62(1):91–8.

16. Goodall J, Mateo J, Yuan W, Mossop H, Porta N, Miranda S, et al. Circulating cell-free DNA to guide prostate cancer treatment with PARP inhibition. Cancer Discov 2017;7(9):1006–17.

17. Zhang M, Liu G, Xue F, Edwards R, Sood AK, Zhang W, et al. Copy number deletion of RAD50 as predictive marker of BRCAness and PARP inhibitor response in BRCA wild type ovarian cancer. Gynecol Oncol 2016;141(1):57–64.

18. Liu Y, Burness ML, Martin-Trevino R, Guy J, Bai S, Harouaka R, et al. RAD51 mediates resistance of cancer stem cells to PARP inhibition in triple-negative breast cancer. Clin Cancer Res 2017;23(2):514–22.

19. Min A, Im SA, Yoon YK, Song SH, Nam HJ, Hur HS, et al. RAD51C-deficient cancer cells are highly sensitive to the PARP inhibitor olaparib. Mol Cancer Ther 2013;12(6):865–77.

20. Loveday C, Turnbull C, Ramsay E, Hughes D, Ruark E, Frankum JR, et al. Germline mutations in RAD51D confer susceptibility to ovarian cancer. Nat Genet 2011;43(9):879–82.

21. Wang J, Ding Q, Fujimori H, Motegi A, Miki Y, Masutani M. Loss of CtIP disturbs homologous recombination repair and sensitizes breast cancer cells to PARP inhibitors. Oncotarget 2016;7(7):7701–14.

22. Lin ZP, Ratner ES, Whicker ME, Lee Y, Sartorelli AC. Triapine disrupts CtIP-mediated homologous recombination repair and sensitizes ovarian cancer cells to PARP and topoisomerase inhibitors. Mol Cancer Res 2014;12(3):381–93.

23. Bryant HE, Schultz N, Thomas HD, Parker KM, Flower D, Lopez E, et al. Specific killing of BRCA2-deficient tumours with inhibitors of poly(ADP-ribose) polymerase. Nature 2005;434(7035):913–7.
